# Supplementary material for: A ten-year review of indications and outcomes of obstetric admissions to an intensive care unit in a low-resource country
Source: PLoS One. 2021 Dec 31;16(12):e0261974. doi: 10.1371/journal.pone.0261974 (PMC8719704; doi:10.1371/journal.pone.0261974)
Supplement: Attachment — Submitted filename: Response to Reviewers.docx [file pone.0261974.s002.docx]

**RESPONSE TO REVIEWER**

1. It would be good to have the abstract separated as introduction, methods, results and conclusion

Response: The abstract has now been structured.

2. Typos
   a. Line 161 - No need for the “:”
   b. Line 222 - No need for the “:”
   c. Line 223 - please do not write STATA It is Stata (see the official Stata documentation).
   d. Line 273 - No need for the “/”
   e. Line 293 - It should be “Medical” not “Med9cal

Response:

- ‘:’ has been deleted in line 166
- ‘:’ has been deleted in line 227
- STATA has been changed to Stata in line 228
- ‘/’ deleted in line 272
- Spelling of medical corrected in line 298

3. Please correct from multivariate to multivariable in the whole document

Response: ‘Multivariate’ have been corrected to ‘multivariable’ in lines 236 and 319

4. In the “Factors influencing outcomes following obstetric ICU admission” subsection, please:
   a. add descriptives of the length of stay. At least min/max and the mean.

Response:

The length of stay, with the minimum and the maximum number of days were already provided under Background and Clinical Characteristics of participants in the Results section in lines 268 to 270. They have now been highlighted in red.

The mean duration of stay has been included in line 270 and also in Table 1 and highlighted.

b. Describe the overall survival as well. Example: that ¼ of the patients died in XX days (Stata sts list command should offer that). In the same line you can extract the median survival

Response:

These have been provided in lines 307 to 310 as ‘About 14.5% (62/428) of study participants died on day 1 of ICU admission, 4.4% (19/428) on day 2 and 2.3% (10/428) on day 3. More than half of the ICU obstetric deaths (53.9%, 62/115) occurred within 24 hours of admission, 16.5% (19/115) on day 2 and 8.7% (10/115) on day 3. The median survival time is 10 days (95% CI 6,14).

5. Table 2 - please add the reference categories. For example, for age we need the “below 25” and as HR we would put “1” or “Reference”.

Response: Table 2 has been revised.

6a. Add an overall survival curve so you would have a), b), c) and d)
Response:

The overall survival curve has been provided as the 4^th^ graph in Figure 4.

All the survival curves have been put under one file and Figure 4 named ‘4a’, Figure 5 named ‘4b’, Figure 6 named ‘4c’, and the overall survival estimates named ‘4d’.

6b. Report the risk set per each 5 day
6c. Please censor time at 15 or 20th day.

**Response**

If 6b and 6c are sub-parts of the same instruction, as we think, then they have been included in lines 334 to 341 as ‘Within the first 5 days, the survivor function was 0.7664 (95%CI 0.7233, 0.8036). In the second 5 days (i.e. days 5-9), survivor function was 0.6282 (95%CI 0.5415, 0.7030). In the third five days (i.e. 10-14), the survivor function was 0.4607 (95%CI 0.3047, 0.6032) and this particular survivor function remained constant over the next grouped days (15-45). Censoring time at day 15, the survivor functions for the first, second and third set of 5 days remained as reported above. However, the survivor function changed for the last set of days (15-45) to 0.2303 (95%CI 0.0208, 0.5733). Censoring did not change the survivor function or risk of outcome in the three sets of 5 days earlier indicated.

7. Line 326 it mentions a supplementary file. I did not see such a file.

Response:

We apologise for this mishap. The line has been deleted because all relevant files have already been provided.
